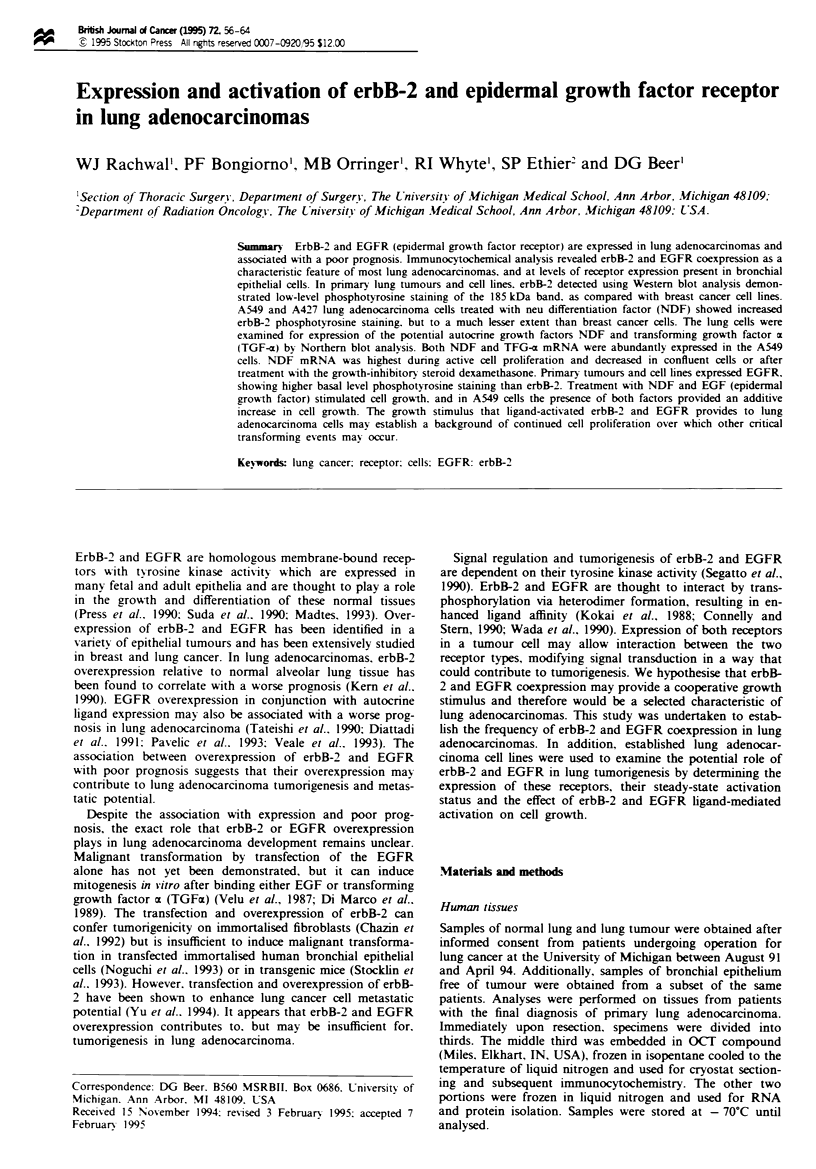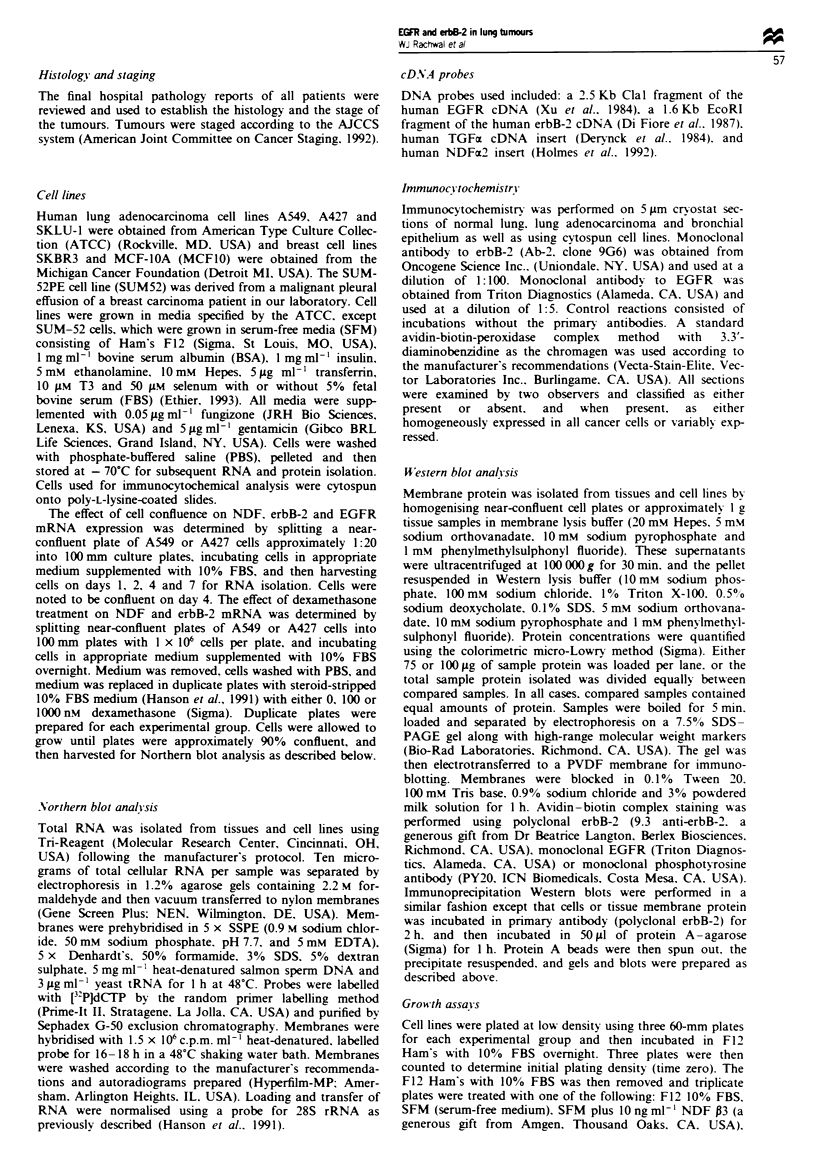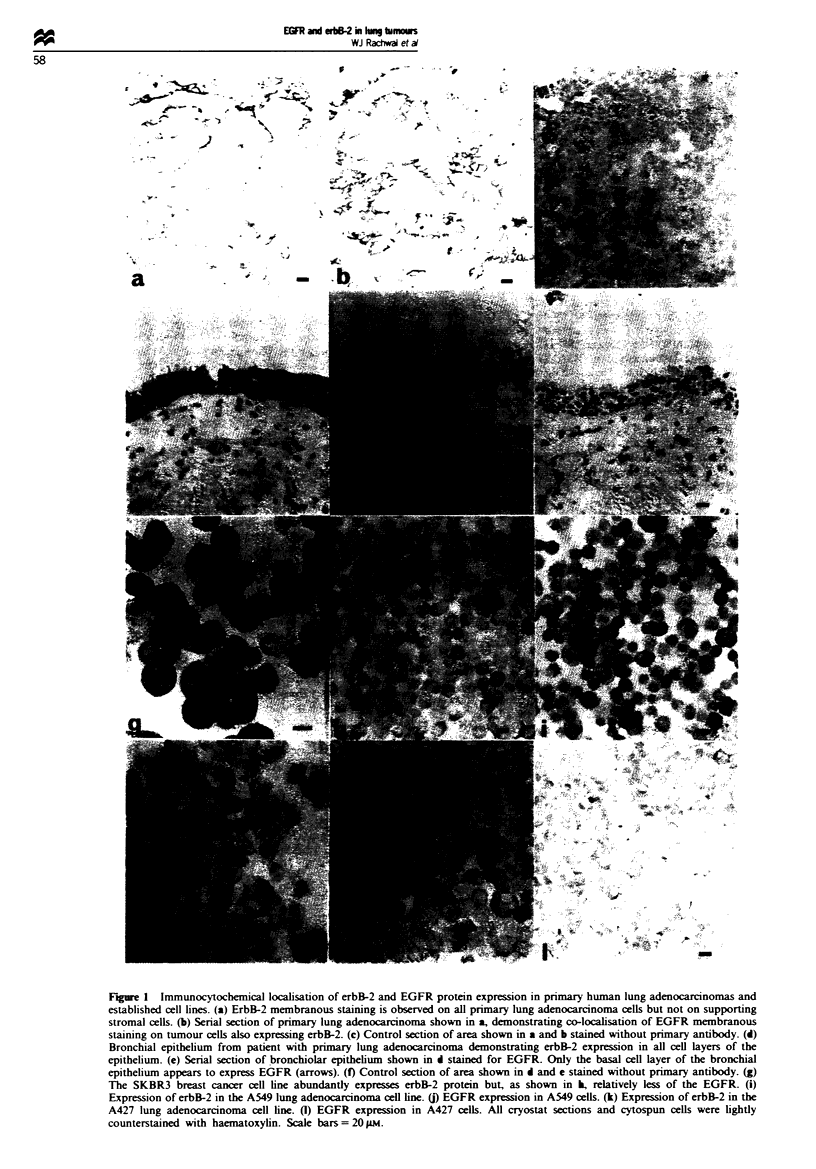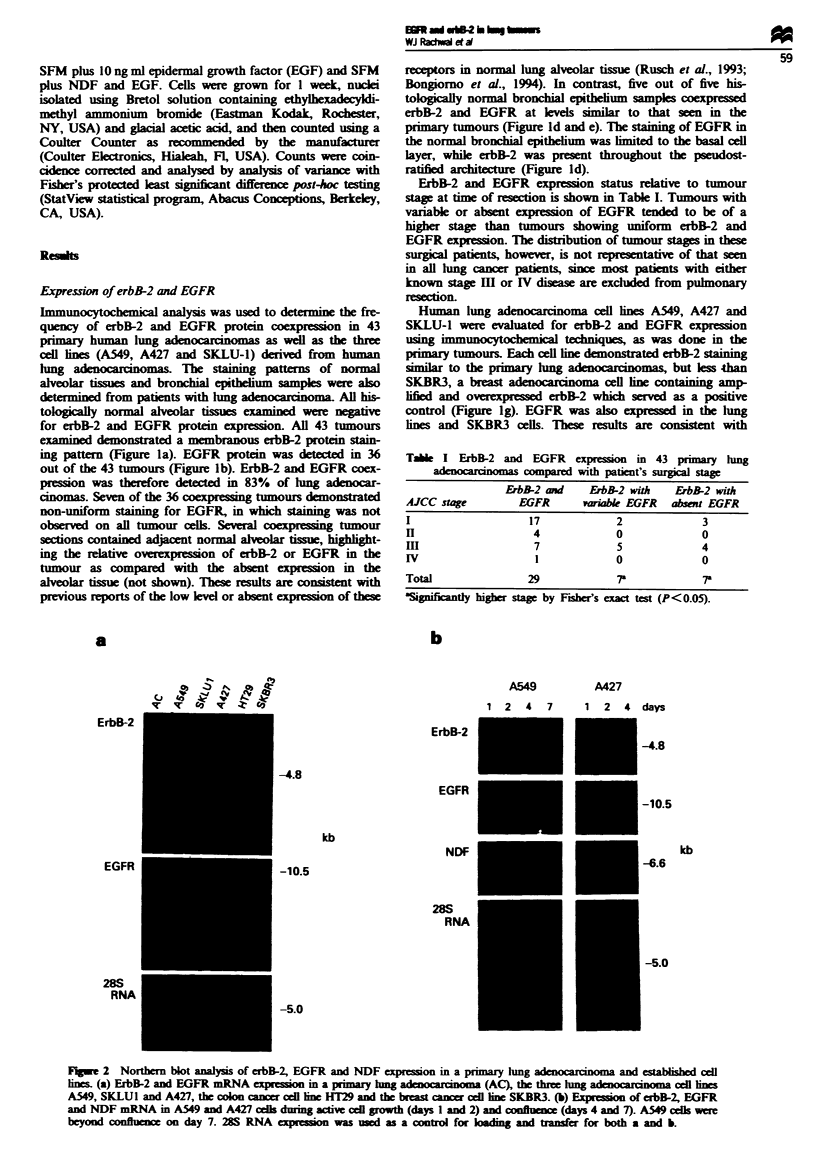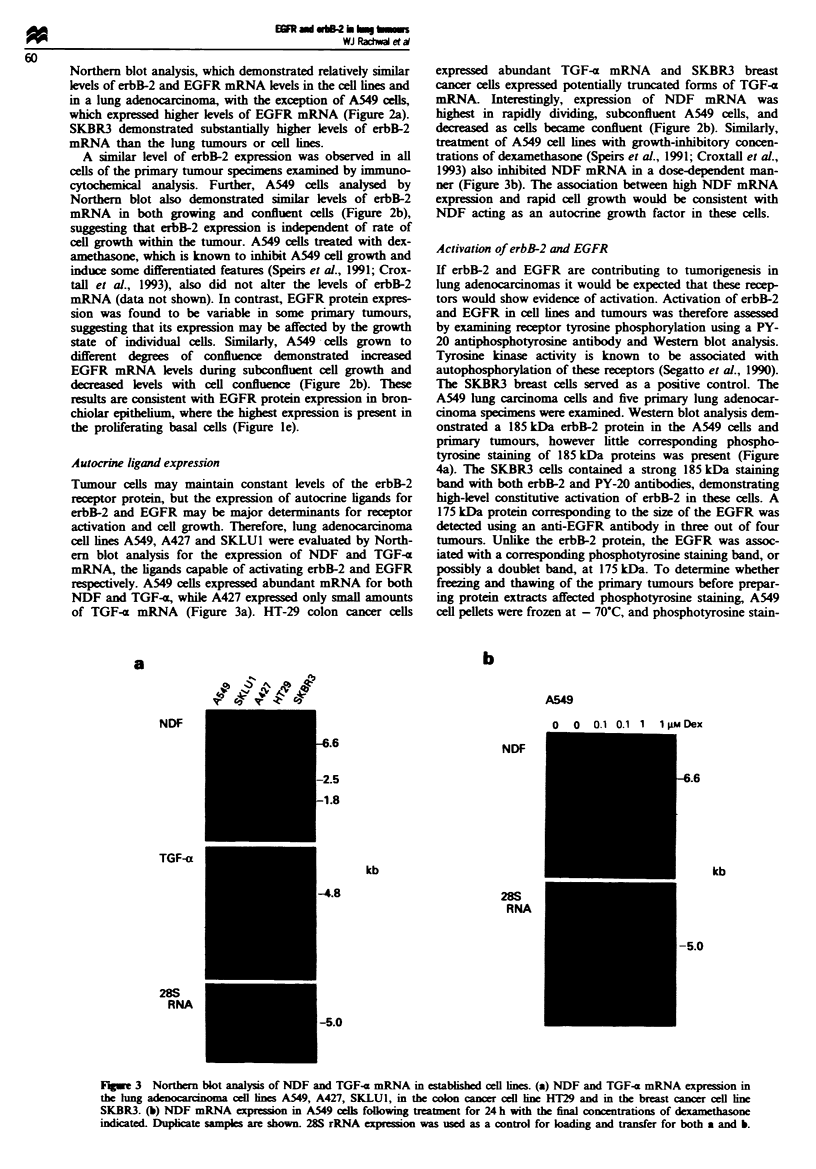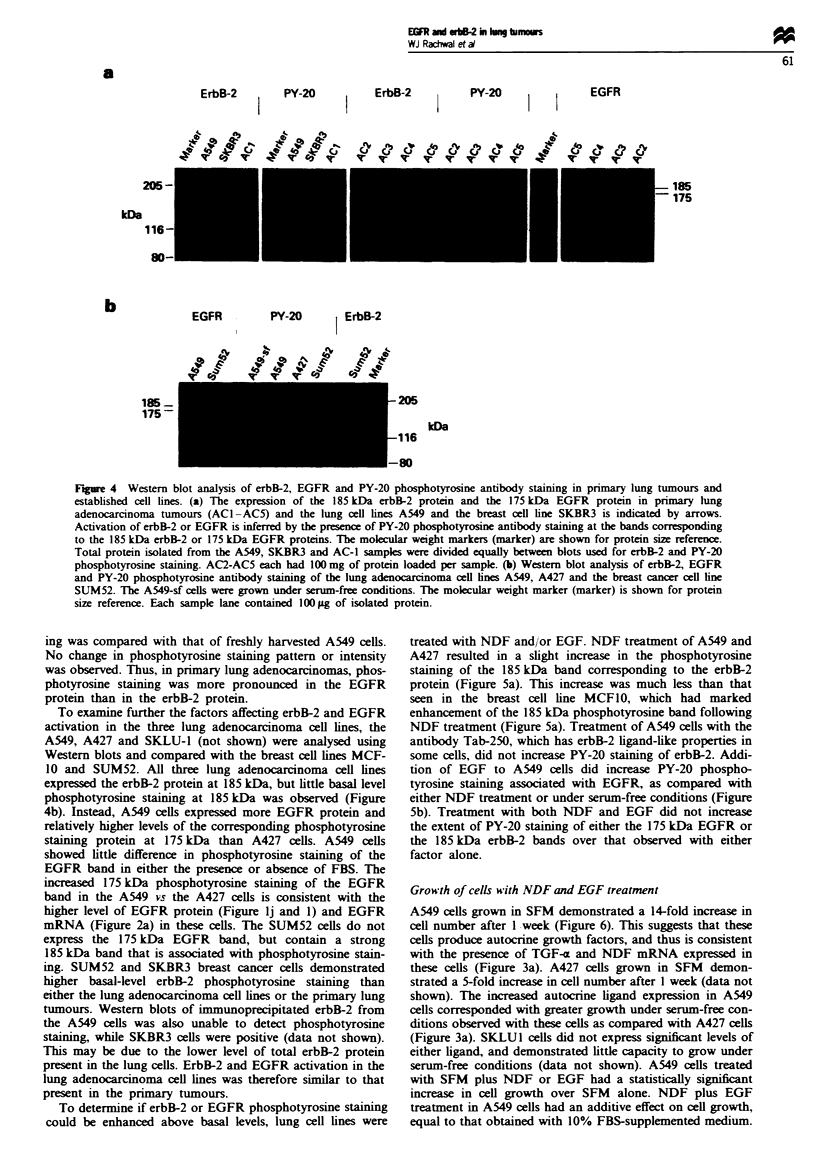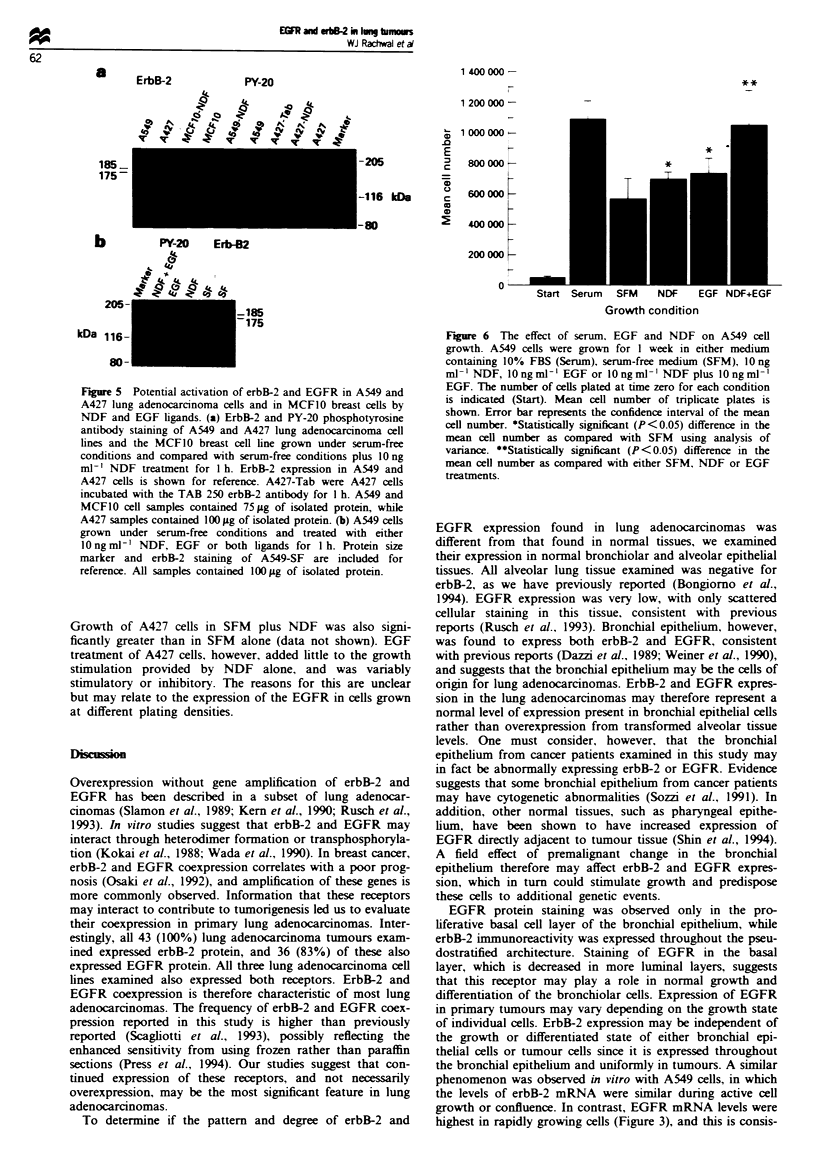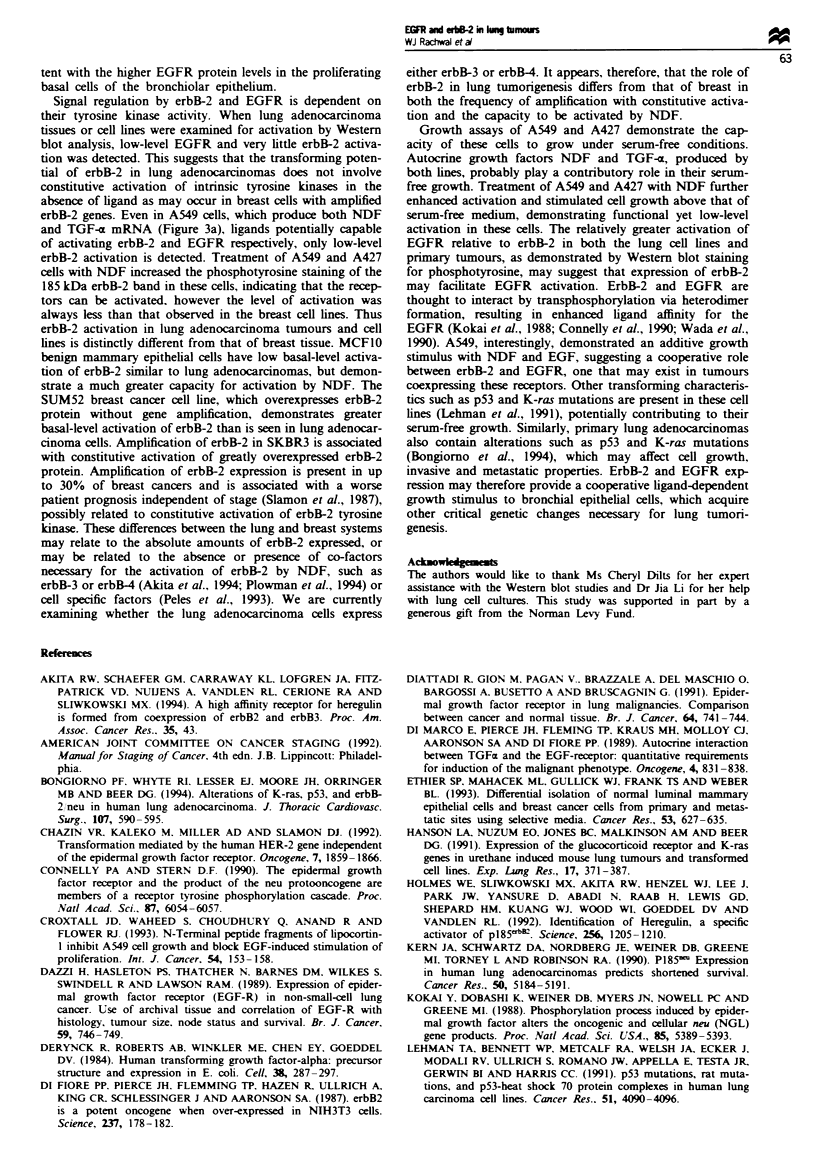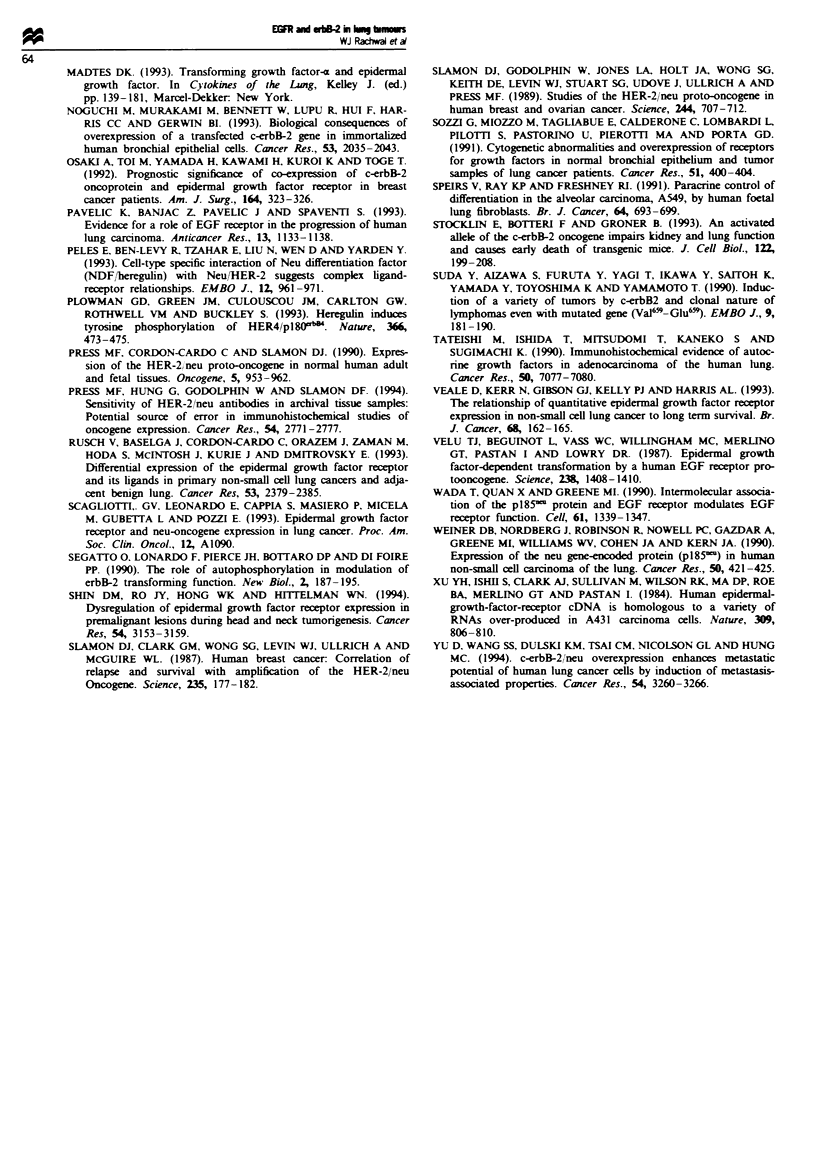# Expression and activation of erbB-2 and epidermal growth factor receptor in lung adenocarcinomas.

**DOI:** 10.1038/bjc.1995.277

**Published:** 1995-07

**Authors:** W. J. Rachwal, P. F. Bongiorno, M. B. Orringer, R. I. Whyte, S. P. Ethier, D. G. Beer

**Affiliations:** Department of Surgery, University of Michigan Medical School, Ann Arbor 48109, USA.

## Abstract

**Images:**


British Journal of Cancer (1995) 72, 56-64

© 1995 Stockton Press All rights reserved 0007-0920/95 $12.00

### Expression and activation of erbB-2 and epidermal growth factor receptor in lung adenocarcinomas

WJ Rachwal[1], PF Bongiorno[1], MB Orringer[1], RI Whyte[1], SP Ethier[2] and DG Beer[1]

[1]*Section of Thoracic Surgery, Department of Surgery, The University of Michigan Medical School, Ann Arbor, Michigan 48109;* [2]*Department of Radiation Oncology, The University of Michigan Medical School, Ann Arbor, Michigan 48109; USA.*

**Summary** ErbB-2 and EGFR (epidermal growth factor receptor) are expressed in lung adenocarcinomas and associated with a poor prognosis. Immunocytochemical analysis revealed erbB-2 and EGFR coexpression as a characteristic feature of most lung adenocarcinomas, and at levels of receptor expression present in bronchial epithelial cells. In primary lung tumours and cell lines, erbB-2 detected using Western blot analysis demonstrated low-level phosphotyrosine staining of the 185 kDa band, as compared with breast cancer cell lines. A549 and A427 lung adenocarcinoma cells treated with neu differentiation factor (NDF) showed increased erbB-2 phosphotyrosine staining, but to a much lesser extent than breast cancer cells. The lung cells were examined for expression of the potential autocrine growth factors NDF and transforming growth factor α (TGF-α) by Northern blot analysis. Both NDF and TFG-α mRNA were abundantly expressed in the A549 cells. NDF mRNA was highest during active cell proliferation and decreased in confluent cells or after treatment with the growth-inhibitory steroid dexamethasone. Primary tumours and cell lines expressed EGFR, showing higher basal level phosphotyrosine staining than erbB-2. Treatment with NDF and EGF (epidermal growth factor) stimulated cell growth, and in A549 cells the presence of both factors provided an additive increase in cell growth. The growth stimulus that ligand-activated erbB-2 and EGFR provides to lung adenocarcinoma cells may establish a background of continued cell proliferation over which other critical transforming events may occur.

**Keywords:** lung cancer; receptor; cells; EGFR; erbB-2

ErbB-2 and EGFR are homologous membrane-bound receptors with tyrosine kinase activity which are expressed in many fetal and adult epithelia and are thought to play a role in the growth and differentiation of these normal tissues (Press *et al*., 1990; Suda *et al*., 1990; Madtes, 1993). Overexpression of erbB-2 and EGFR has been identified in a variety of epithelial tumours and has been extensively studied in breast and lung cancer. In lung adenocarcinomas, erbB-2 overexpression relative to normal alveolar lung tissue has been found to correlate with a worse prognosis (Kern *et al*., 1990). EGFR overexpression in conjunction with autocrine ligand expression may also be associated with a worse prognosis in lung adenocarcinoma (Tateishi *et al*., 1990; Diattadi *et al*., 1991; Pavelic *et al*., 1993; Veale *et al*., 1993). The association between overexpression of erbB-2 and EGFR with poor prognosis suggests that their overexpression may contribute to lung adenocarcinoma tumorigenesis and metastatic potential.

Despite the association with expression and poor prognosis, the exact role that erbB-2 or EGFR overexpression plays in lung adenocarcinoma development remains unclear. Malignant transformation by transfection of the EGFR alone has not yet been demonstrated, but it can induce mitogenesis *in vitro* after binding either EGF or transforming growth factor α (TGFα) (Velu *et al*., 1987; Di Marco *et al*., 1989). The transfection and overexpression of erbB-2 can confer tumorigenicity on immortalised fibroblasts (Chazin *et al*., 1992) but is insufficient to induce malignant transformation in transfected immortalised human bronchial epithelial cells (Noguchi *et al*., 1993) or in transgenic mice (Stocklin *et al*., 1993). However, transfection and overexpression of erbB-2 have been shown to enhance lung cancer cell metastatic potential (Yu *et al*., 1994). It appears that erbB-2 and EGFR overexpression contributes to, but may be insufficient for, tumorigenesis in lung adenocarcinoma.

Correspondence: DG Beer, B560 MSRBII, Box 0686, University of Michigan, Ann Arbor, MI 48109, USA

Received 15 November 1994; revised 3 February 1995; accepted 7 February 1995

Signal regulation and tumorigenesis of erbB-2 and EGFR are dependent on their tyrosine kinase activity (Segatto *et al*., 1990). ErbB-2 and EGFR are thought to interact by transphosphorylation via heterodimer formation, resulting in enhanced ligand affinity (Kokai *et al*., 1988; Connelly and Stern, 1990; Wada *et al*., 1990). Expression of both receptors in a tumour cell may allow interaction between the two receptor types, modifying signal transduction in a way that could contribute to tumorigenesis. We hypothesise that erbB-2 and EGFR coexpression may provide a cooperative growth stimulus and therefore would be a selected characteristic of lung adenocarcinomas. This study was undertaken to establish the frequency of erbB-2 and EGFR coexpression in lung adenocarcinomas. In addition, established lung adenocarcinoma cell lines were used to examine the potential role of erbB-2 and EGFR in lung tumorigenesis by determining the expression of these receptors, their steady-state activation status and the effect of erbB-2 and EGFR ligand-mediated activation on cell growth.

### Materials and methods

### *Human tissues*

Samples of normal lung and lung tumour were obtained after informed consent from patients undergoing operation for lung cancer at the University of Michigan between August 91 and April 94. Additionally, samples of bronchial epithelium free of tumour were obtained from a subset of the same patients. Analyses were performed on tissues from patients with the final diagnosis of primary lung adenocarcinoma. Immediately upon resection, specimens were divided into thirds. The middle third was embedded in OCT compound (Miles, Elkhart, IN, USA), frozen in isopentane cooled to the temperature of liquid nitrogen and used for cryostat sectioning and subsequent immunocytochemistry. The other two portions were frozen in liquid nitrogen and used for RNA and protein isolation. Samples were stored at − 70°C until analysed.

*Histology and staging*

The final hospital pathology reports of all patients were reviewed and used to establish the histology and the stage of the tumours. Tumours were staged according to the AJCCS system (American Joint Committee on Cancer Staging, 1992).

*Cell lines*

Human lung adenocarcinoma cell lines A549, A427 and SKLU-1 were obtained from American Type Culture Collection (ATCC) (Rockville, MD, USA) and breast cell lines SKBR3 and MCF-10A (MCF10) were obtained from the Michigan Cancer Foundation (Detroit MI, USA). The SUM-52PE cell line (SUM52) was derived from a malignant pleural effusion of a breast carcinoma patient in our laboratory. Cell lines were grown in media specified by the ATCC, except SUM-52 cells, which were grown in serum-free media (SFM) consisting of Ham's F12 (Sigma, St Louis, MO, USA), 1 mg $ml^{-1}$ bovine serum albumin (BSA), 1 mg $ml^{-1}$ insulin, 5 mM ethanolamine, 10 mM Hepes, 5 μg $ml^{-1}$ transferrin, 10 μM T3 and 50 μM selenum with or without 5% fetal bovine serum (FBS) (Ethier, 1993). All media were supplemented with 0.05 μg $ml^{-1}$ fungizone (JRH Bio Sciences, Lenexa, KS, USA) and 5 μg $ml^{-1}$ gentamicin (Gibco BRL Life Sciences, Grand Island, NY, USA). Cells were washed with phosphate-buffered saline (PBS), pelleted and then stored at − 70°C for subsequent RNA and protein isolation. Cells used for immunocytochemical analysis were cytospun onto poly-L-lysine-coated slides.

The effect of cell confluence on NDF, erbB-2 and EGFR mRNA expression was determined by splitting a near-confluent plate of A549 or A427 cells approximately 1:20 into 100 mm culture plates, incubating cells in appropriate medium supplemented with 10% FBS, and then harvesting cells on days 1, 2, 4 and 7 for RNA isolation. Cells were noted to be confluent on day 4. The effect of dexamethasone treatment on NDF and erbB-2 mRNA was determined by splitting near-confluent plates of A549 or A427 cells into 100 mm plates with $1 \times 10^6$ cells per plate, and incubating cells in appropriate medium supplemented with 10% FBS overnight. Medium was removed, cells washed with PBS, and medium was replaced in duplicate plates with steroid-stripped 10% FBS medium (Hanson *et al.*, 1991) with either 0, 100 or 1000 nM dexamethasone (Sigma). Duplicate plates were prepared for each experimental group. Cells were allowed to grow until plates were approximately 90% confluent, and then harvested for Northern blot analysis as described below.

*Northern blot analysis*

Total RNA was isolated from tissues and cell lines using Tri-Reagent (Molecular Research Center, Cincinnati, OH, USA) following the manufacturer's protocol. Ten micrograms of total cellular RNA per sample was separated by electrophoresis in 1.2% agarose gels containing 2.2 M formaldehyde and then vacuum transferred to nylon membranes (Gene Screen Plus; NEN, Wilmington, DE, USA). Membranes were prehybridised in 5 × SSPE (0.9 M sodium chloride, 50 mM sodium phosphate, pH 7.7, and 5 mM EDTA), 5 × Denhardt's, 50% formamide, 3% SDS, 5% dextran sulphate, 5 mg $ml^{-1}$ heat-denatured salmon sperm DNA and 3 μg $ml^{-1}$ yeast tRNA for 1 h at 48°C. Probes were labelled with [$^{32}$P]dCTP by the random primer labelling method (Prime-It II, Stratagene, La Jolla, CA, USA) and purified by Sephadex G-50 exclusion chromatography. Membranes were hybridised with $1.5 \times 10^6$ c.p.m. $ml^{-1}$ heat-denatured, labelled probe for 16–18 h in a 48°C shaking water bath. Membranes were washed according to the manufacturer's recommendations and autoradiograms prepared (Hyperfilm-MP; Amersham, Arlington Heights, IL, USA). Loading and transfer of RNA were normalised using a probe for 28S rRNA as previously described (Hanson *et al.*, 1991).

*cDNA probes*

DNA probes used included: a 2.5 Kb ClaI fragment of the human EGFR cDNA (Xu *et al.*, 1984), a 1.6 Kb EcoRI fragment of the human erbB-2 cDNA (Di Fiore *et al.*, 1987), human TGFα cDNA insert (Derynck *et al.*, 1984), and human NDFα2 insert (Holmes *et al.*, 1992).

*Immunocytochemistry*

Immunocytochemistry was performed on 5 μm cryostat sections of normal lung, lung adenocarcinoma and bronchial epithelium as well as using cytospun cell lines. Monoclonal antibody to erbB-2 (Ab-2, clone 9G6) was obtained from Oncogene Science Inc., (Uniondale, NY, USA) and used at a dilution of 1:100. Monoclonal antibody to EGFR was obtained from Triton Diagnostics (Alameda, CA, USA) and used at a dilution of 1:5. Control reactions consisted of incubations without the primary antibodies. A standard avidin-biotin-peroxidase complex method with 3,3′-diaminobenzidine as the chromagen was used according to the manufacturer's recommendations (Vecta-Stain-Elite, Vector Laboratories Inc., Burlingame, CA, USA). All sections were examined by two observers and classified as either present or absent, and when present, as either homogeneously expressed in all cancer cells or variably expressed.

*Western blot analysis*

Membrane protein was isolated from tissues and cell lines by homogenising near-confluent cell plates or approximately 1 g tissue samples in membrane lysis buffer (20 mM Hepes, 5 mM sodium orthovanadate, 10 mM sodium pyrophosphate and 1 mM phenylmethylsulphonyl fluoride). These supernatants were ultracentrifuged at 100 000 *g* for 30 min, and the pellet resuspended in Western lysis buffer (10 mM sodium phosphate, 100 mM sodium chloride, 1% Triton X-100, 0.5% sodium deoxycholate, 0.1% SDS, 5 mM sodium orthovanadate, 10 mM sodium pyrophosphate and 1 mM phenylmethylsulphonyl fluoride). Protein concentrations were quantified using the colorimetric micro-Lowry method (Sigma). Either 75 or 100 μg of sample protein was loaded per lane, or the total sample protein isolated was divided equally between compared samples. In all cases, compared samples contained equal amounts of protein. Samples were boiled for 5 min, loaded and separated by electrophoresis on a 7.5% SDS–PAGE gel along with high-range molecular weight markers (Bio-Rad Laboratories, Richmond, CA, USA). The gel was then electrotransferred to a PVDF membrane for immunoblotting. Membranes were blocked in 0.1% Tween 20, 100 mM Tris base, 0.9% sodium chloride and 3% powdered milk solution for 1 h. Avidin–biotin complex staining was performed using polyclonal erbB-2 (9.3 anti-erbB-2, a generous gift from Dr Beatrice Langton, Berlex Biosciences, Richmond, CA, USA), monoclonal EGFR (Triton Diagnostics, Alameda, CA, USA) or monoclonal phosphotyrosine antibody (PY20, ICN Biomedicals, Costa Mesa, CA, USA). Immunoprecipitation Western blots were performed in a similar fashion except that cells or tissue membrane protein was incubated in primary antibody (polyclonal erbB-2) for 2 h, and then incubated in 50 μl of protein A–agarose (Sigma) for 1 h. Protein A beads were then spun out, the precipitate resuspended, and gels and blots were prepared as described above.

*Growth assays*

Cell lines were plated at low density using three 60-mm plates for each experimental group and then incubated in F12 Ham's with 10% FBS overnight. Three plates were then counted to determine initial plating density (time zero). The F12 Ham's with 10% FBS was then removed and triplicate plates were treated with one of the following: F12 10% FBS, SFM (serum-free medium), SFM plus 10 ng $ml^{-1}$ NDF β3 (a generous gift from Amgen, Thousand Oaks, CA, USA),

**Figure 1** Immunocytochemical localisation of erbB-2 and EGFR protein expression in primary human lung adenocarcinomas and established cell lines. (**a**) ErbB-2 membranous staining is observed on all primary lung adenocarcinoma cells but not on supporting stromal cells. (**b**) Serial section of primary lung adenocarcinoma shown in **a**, demonstrating co-localisation of EGFR membranous staining on tumour cells also expressing erbB-2. (**c**) Control section of area shown in **a** and **b** stained without primary antibody. (**d**) Bronchial epithelium from patient with primary lung adenocarcinoma demonstrating erbB-2 expression in all cell layers of the epithelium. (**e**) Serial section of bronchiolar epithelium shown in **d** stained for EGFR. Only the basal cell layer of the bronchial epithelium appears to express EGFR (arrows). (**f**) Control section of area shown in **d** and **e** stained without primary antibody. (**g**) The SKBR3 breast cancer cell line abundantly expresses erbB-2 protein but, as shown in **h**, relatively less of the EGFR. (**i**) Expression of erbB-2 in the A549 lung adenocarcinoma cell line. (**j**) EGFR expression in A549 cells. (**k**) Expression of erbB-2 in the A427 lung adenocarcinoma cell line. (**l**) EGFR expression in A427 cells. All cryostat sections and cytospun cells were lightly counterstained with haematoxylin. Scale bars = 20 μM.

SFM plus 10 ng ml epidermal growth factor (EGF) and SFM plus NDF and EGF. Cells were grown for 1 week, nuclei isolated using Bretol solution containing ethylhexadecyldimethyl ammonium bromide (Eastman Kodak, Rochester, NY, USA) and glacial acetic acid, and then counted using a Coulter Counter as recommended by the manufacturer (Coulter Electronics, Hialeah, Fl, USA). Counts were coincidence corrected and analysed by analysis of variance with Fisher's protected least significant difference *post-hoc* testing (StatView statistical program, Abacus Conceptions, Berkeley, CA, USA).

### Results

### *Expression of erbB-2 and EGFR*

Immunocytochemical analysis was used to determine the frequency of erbB-2 and EGFR protein coexpression in 43 primary human lung adenocarcinomas as well as the three cell lines (A549, A427 and SKLU-1) derived from human lung adenocarcinomas. The staining patterns of normal alveolar tissues and bronchial epithelium samples were also determined from patients with lung adenocarcinoma. All histologically normal alveolar tissues examined were negative for erbB-2 and EGFR protein expression. All 43 tumours examined demonstrated a membranous erbB-2 protein staining pattern (Figure 1a). EGFR protein was detected in 36 out of the 43 tumours (Figure 1b). ErbB-2 and EGFR coexpression was therefore detected in 83% of lung adenocarcinomas. Seven of the 36 coexpressing tumours demonstrated non-uniform staining for EGFR, in which staining was not observed on all tumour cells. Several coexpressing tumour sections contained adjacent normal alveolar tissue, highlighting the relative overexpression of erbB-2 or EGFR in the tumour as compared with the absent expression in the alveolar tissue (not shown). These results are consistent with previous reports of the low level or absent expression of these receptors in normal lung alveolar tissue (Rusch *et al*., 1993; Bongiorno *et al*., 1994). In contrast, five out of five histologically normal bronchial epithelium samples coexpressed erbB-2 and EGFR at levels similar to that seen in the primary tumours (Figure 1d and e). The staining of EGFR in the normal bronchial epithelium was limited to the basal cell layer, while erbB-2 was present throughout the pseudostratified architecture (Figure 1d).

ErbB-2 and EGFR expression status relative to tumour stage at time of resection is shown in Table I. Tumours with variable or absent expression of EGFR tended to be of a higher stage than tumours showing uniform erbB-2 and EGFR expression. The distribution of tumour stages in these surgical patients, however, is not representative of that seen in all lung cancer patients, since most patients with either known stage III or IV disease are excluded from pulmonary resection.

Human lung adenocarcinoma cell lines A549, A427 and SKLU-1 were evaluated for erbB-2 and EGFR expression using immunocytochemical techniques, as was done in the primary tumours. Each cell line demonstrated erbB-2 staining similar to the primary lung adenocarcinomas, but less than SKBR3, a breast adenocarcinoma cell line containing amplified and overexpressed erbB-2 which served as a positive control (Figure 1g). EGFR was also expressed in the lung lines and SKBR3 cells. These results are consistent with

**Table I** ErbB-2 and EGFR expression in 43 primary lung adenocarcinomas compared with patient's surgical stage

| *AJCC stage* | *ErbB-2 and EGFR* | *ErbB-2 with variable EGFR* | *ErbB-2 with absent EGFR* |
|---|---|---|---|
| I | 17 | 2 | 3 |
| II | 4 | 0 | 0 |
| III | 7 | 5 | 4 |
| IV | 1 | 0 | 0 |
| Total | 29 | 7[a] | 7[a] |

[a]Significantly higher stage by Fisher's exact test ($P<0.05$).

**Figure 2** Northern blot analysis of erbB-2, EGFR and NDF expression in a primary lung adenocarcinoma and established cell lines. (**a**) ErbB-2 and EGFR mRNA expression in a primary lung adenocarcinoma (AC), the three lung adenocarcinoma cell lines A549, SKLU1 and A427, the colon cancer cell line HT29 and the breast cancer cell line SKBR3. (**b**) Expression of erbB-2, EGFR and NDF mRNA in A549 and A427 cells during active cell growth (days 1 and 2) and confluence (days 4 and 7). A549 cells were beyond confluence on day 7. 28S RNA expression was used as a control for loading and transfer for both **a** and **b**.

Northern blot analysis, which demonstrated relatively similar levels of erbB-2 and EGFR mRNA levels in the cell lines and in a lung adenocarcinoma, with the exception of A549 cells, which expressed higher levels of EGFR mRNA (Figure 2a). SKBR3 demonstrated substantially higher levels of erbB-2 mRNA than the lung tumours or cell lines.

A similar level of erbB-2 expression was observed in all cells of the primary tumour specimens examined by immunocytochemical analysis. Further, A549 cells analysed by Northern blot also demonstrated similar levels of erbB-2 mRNA in both growing and confluent cells (Figure 2b), suggesting that erbB-2 expression is independent of rate of cell growth within the tumour. A549 cells treated with dexamethasone, which is known to inhibit A549 cell growth and induce some differentiated features (Speirs *et al.*, 1991; Croxtall *et al.*, 1993), also did not alter the levels of erbB-2 mRNA (data not shown). In contrast, EGFR protein expression was found to be variable in some primary tumours, suggesting that its expression may be affected by the growth state of individual cells. Similarly, A549 cells grown to different degrees of confluence demonstrated increased EGFR mRNA levels during subconfluent cell growth and decreased levels with cell confluence (Figure 2b). These results are consistent with EGFR protein expression in bronchiolar epithelium, where the highest expression is present in the proliferating basal cells (Figure 1e).

### *Autocrine ligand expression*

Tumour cells may maintain constant levels of the erbB-2 receptor protein, but the expression of autocrine ligands for erbB-2 and EGFR may be major determinants for receptor activation and cell growth. Therefore, lung adenocarcinoma cell lines A549, A427 and SKLU1 were evaluated by Northern blot analysis for the expression of NDF and TGF-α mRNA, the ligands capable of activating erbB-2 and EGFR respectively. A549 cells expressed abundant mRNA for both NDF and TGF-α, while A427 expressed only small amounts of TGF-α mRNA (Figure 3a). HT-29 colon cancer cells expressed abundant TGF-α mRNA and SKBR3 breast cancer cells expressed potentially truncated forms of TGF-α mRNA. Interestingly, expression of NDF mRNA was highest in rapidly dividing, subconfluent A549 cells, and decreased as cells became confluent (Figure 2b). Similarly, treatment of A549 cell lines with growth-inhibitory concentrations of dexamethasone (Speirs *et al.*, 1991; Croxtall *et al.*, 1993) also inhibited NDF mRNA in a dose-dependent manner (Figure 3b). The association between high NDF mRNA expression and rapid cell growth would be consistent with NDF acting as an autocrine growth factor in these cells.

### *Activation of erbB-2 and EGFR*

If erbB-2 and EGFR are contributing to tumorigenesis in lung adenocarcinomas it would be expected that these receptors would show evidence of activation. Activation of erbB-2 and EGFR in cell lines and tumours was therefore assessed by examining receptor tyrosine phosphorylation using a PY-20 antiphosphotyrosine antibody and Western blot analysis. Tyrosine kinase activity is known to be associated with autophosphorylation of these receptors (Segatto *et al.*, 1990). The SKBR3 breast cells served as a positive control. The A549 lung carcinoma cells and five primary lung adenocarcinoma specimens were examined. Western blot analysis demonstrated a 185 kDa erbB-2 protein in the A549 cells and primary tumours, however little corresponding phosphotyrosine staining of 185 kDa proteins was present (Figure 4a). The SKBR3 cells contained a strong 185 kDa staining band with both erbB-2 and PY-20 antibodies, demonstrating high-level constitutive activation of erbB-2 in these cells. A 175 kDa protein corresponding to the size of the EGFR was detected using an anti-EGFR antibody in three out of four tumours. Unlike the erbB-2 protein, the EGFR was associated with a corresponding phosphotyrosine staining band, or possibly a doublet band, at 175 kDa. To determine whether freezing and thawing of the primary tumours before preparing protein extracts affected phosphotyrosine staining, A549 cell pellets were frozen at − 70°C, and phosphotyrosine stain-

**Figure 3** Northern blot analysis of NDF and TGF-α mRNA in established cell lines. (**a**) NDF and TGF-α mRNA expression in the lung adenocarcinoma cell lines A549, A427, SKLU1, in the colon cancer cell line HT29 and in the breast cancer cell line SKBR3. (**b**) NDF mRNA expression in A549 cells following treatment for 24 h with the final concentrations of dexamethasone indicated. Duplicate samples are shown. 28S rRNA expression was used as a control for loading and transfer for both **a** and **b**.

**Figure 4** Western blot analysis of erbB-2, EGFR and PY-20 phosphotyrosine antibody staining in primary lung tumours and established cell lines. (**a**) The expression of the 185 kDa erbB-2 protein and the 175 kDa EGFR protein in primary lung adenocarcinoma tumours (AC1–AC5) and the lung cell lines A549 and the breast cell line SKBR3 is indicated by arrows. Activation of erbB-2 or EGFR is inferred by the presence of PY-20 phosphotyrosine antibody staining at the bands corresponding to the 185 kDa erbB-2 or 175 kDa EGFR proteins. The molecular weight markers (marker) are shown for protein size reference. Total protein isolated from the A549, SKBR3 and AC-1 samples were divided equally between blots used for erbB-2 and PY-20 phosphotyrosine staining. AC2-AC5 each had 100 mg of protein loaded per sample. (**b**) Western blot analysis of erbB-2, EGFR and PY-20 phosphotyrosine antibody staining of the lung adenocarcinoma cell lines A549, A427 and the breast cancer cell line SUM52. The A549-sf cells were grown under serum-free conditions. The molecular weight marker (marker) is shown for protein size reference. Each sample lane contained 100 μg of isolated protein.

ing was compared with that of freshly harvested A549 cells. No change in phosphotyrosine staining pattern or intensity was observed. Thus, in primary lung adenocarcinomas, phosphotyrosine staining was more pronounced in the EGFR protein than in the erbB-2 protein.

To examine further the factors affecting erbB-2 and EGFR activation in the three lung adenocarcinoma cell lines, the A549, A427 and SKLU-1 (not shown) were analysed using Western blots and compared with the breast cell lines MCF-10 and SUM52. All three lung adenocarcinoma cell lines expressed the erbB-2 protein at 185 kDa, but little basal level phosphotyrosine staining at 185 kDa was observed (Figure 4b). Instead, A549 cells expressed more EGFR protein and relatively higher levels of the corresponding phosphotyrosine staining protein at 175 kDa than A427 cells. A549 cells showed little difference in phosphotyrosine staining of the EGFR band in either the presence or absence of FBS. The increased 175 kDa phosphotyrosine staining of the EGFR band in the A549 *vs* the A427 cells is consistent with the higher level of EGFR protein (Figure 1j and 1) and EGFR mRNA (Figure 2a) in these cells. The SUM52 cells do not express the 175 kDa EGFR band, but contain a strong 185 kDa band that is associated with phosphotyrosine staining. SUM52 and SKBR3 breast cancer cells demonstrated higher basal-level erbB-2 phosphotyrosine staining than either the lung adenocarcinoma cell lines or the primary lung tumours. Western blots of immunoprecipitated erbB-2 from the A549 cells was also unable to detect phosphotyrosine staining, while SKBR3 cells were positive (data not shown). This may be due to the lower level of total erbB-2 protein present in the lung cells. ErbB-2 and EGFR activation in the lung adenocarcinoma cell lines was therefore similar to that present in the primary tumours.

To determine if erbB-2 or EGFR phosphotyrosine staining could be enhanced above basal levels, lung cell lines were treated with NDF and/or EGF. NDF treatment of A549 and A427 resulted in a slight increase in the phosphotyrosine staining of the 185 kDa band corresponding to the erbB-2 protein (Figure 5a). This increase was much less than that seen in the breast cell line MCF10, which had marked enhancement of the 185 kDa phosphotyrosine band following NDF treatment (Figure 5a). Treatment of A549 cells with the antibody Tab-250, which has erbB-2 ligand-like properties in some cells, did not increase PY-20 staining of erbB-2. Addition of EGF to A549 cells did increase PY-20 phosphotyrosine staining associated with EGFR, as compared with either NDF treatment or under serum-free conditions (Figure 5b). Treatment with both NDF and EGF did not increase the extent of PY-20 staining of either the 175 kDa EGFR or the 185 kDa erbB-2 bands over that observed with either factor alone.

### *Growth of cells with NDF and EGF treatment*

A549 cells grown in SFM demonstrated a 14-fold increase in cell number after 1 week (Figure 6). This suggests that these cells produce autocrine growth factors, and thus is consistent with the presence of TGF-α and NDF mRNA expressed in these cells (Figure 3a). A427 cells grown in SFM demonstrated a 5-fold increase in cell number after 1 week (data not shown). The increased autocrine ligand expression in A549 cells corresponded with greater growth under serum-free conditions observed with these cells as compared with A427 cells (Figure 3a). SKLU1 cells did not express significant levels of either ligand, and demonstrated little capacity to grow under serum-free conditions (data not shown). A549 cells treated with SFM plus NDF or EGF had a statistically significant increase in cell growth over SFM alone. NDF plus EGF treatment in A549 cells had an additive effect on cell growth, equal to that obtained with 10% FBS-supplemented medium.

**Figure 5** Potential activation of erbB-2 and EGFR in A549 and A427 lung adenocarcinoma cells and in MCF10 breast cells by NDF and EGF ligands. (**a**) ErbB-2 and PY-20 phosphotyrosine antibody staining of A549 and A427 lung adenocarcinoma cell lines and the MCF10 breast cell line grown under serum-free conditions and compared with serum-free conditions plus 10 ng $ml^{-1}$ NDF treatment for 1 h. ErbB-2 expression in A549 and A427 cells is shown for reference. A427-Tab were A427 cells incubated with the TAB 250 erbB-2 antibody for 1 h. A549 and MCF10 cell samples contained 75 μg of isolated protein, while A427 samples contained 100 μg of isolated protein. (**b**) A549 cells grown under serum-free conditions and treated with either 10 ng $ml^{-1}$ NDF, EGF or both ligands for 1 h. Protein size marker and erbB-2 staining of A549-SF are included for reference. All samples contained 100 μg of isolated protein.

**Figure 6** The effect of serum, EGF and NDF on A549 cell growth. A549 cells were grown for 1 week in either medium containing 10% FBS (Serum), serum-free medium (SFM), 10 ng $ml^{-1}$ NDF, 10 ng $ml^{-1}$ EGF or 10 ng $ml^{-1}$ NDF plus 10 ng $ml^{-1}$ EGF. The number of cells plated at time zero for each condition is indicated (Start). Mean cell number of triplicate plates is shown. Error bar represents the confidence interval of the mean cell number. *Statistically significant ($P<0.05$) difference in the mean cell number as compared with SFM using analysis of variance. **Statistically significant ($P<0.05$) difference in the mean cell number as compared with either SFM, NDF or EGF treatments.

Growth of A427 cells in SFM plus NDF was also significantly greater than in SFM alone (data not shown). EGF treatment of A427 cells, however, added little to the growth stimulation provided by NDF alone, and was variably stimulatory or inhibitory. The reasons for this are unclear but may relate to the expression of the EGFR in cells grown at different plating densities.

### Discussion

Overexpression without gene amplification of erbB-2 and EGFR has been described in a subset of lung adenocarcinomas (Slamon *et al.*, 1989; Kern *et al.*, 1990; Rusch *et al.*, 1993). *In vitro* studies suggest that erbB-2 and EGFR may interact through heterodimer formation or transphosphorylation (Kokai *et al.*, 1988; Wada *et al.*, 1990). In breast cancer, erbB-2 and EGFR coexpression correlates with a poor prognosis (Osaki *et al.*, 1992), and amplification of these genes is more commonly observed. Information that these receptors may interact to contribute to tumorigenesis led us to evaluate their coexpression in primary lung adenocarcinomas. Interestingly, all 43 (100%) lung adenocarcinoma tumours examined expressed erbB-2 protein, and 36 (83%) of these also expressed EGFR protein. All three lung adenocarcinoma cell lines examined also expressed both receptors. ErbB-2 and EGFR coexpression is therefore characteristic of most lung adenocarcinomas. The frequency of erbB-2 and EGFR coexpression reported in this study is higher than previously reported (Scagliotti *et al.*, 1993), possibly reflecting the enhanced sensitivity from using frozen rather than paraffin sections (Press *et al.*, 1994). Our studies suggest that continued expression of these receptors, and not necessarily overexpression, may be the most significant feature in lung adenocarcinomas.

To determine if the pattern and degree of erbB-2 and EGFR expression found in lung adenocarcinomas was different from that found in normal tissues, we examined their expression in normal bronchiolar and alveolar epithelial tissues. All alveolar lung tissue examined was negative for erbB-2, as we have previously reported (Bongiorno *et al.*, 1994). EGFR expression was very low, with only scattered cellular staining in this tissue, consistent with previous reports (Rusch *et al.*, 1993). Bronchial epithelium, however, was found to express both erbB-2 and EGFR, consistent with previous reports (Dazzi *et al.*, 1989; Weiner *et al.*, 1990), and suggests that the bronchial epithelium may be the cells of origin for lung adenocarcinomas. ErbB-2 and EGFR expression in the lung adenocarcinomas may therefore represent a normal level of expression present in bronchial epithelial cells rather than overexpression from transformed alveolar tissue levels. One must consider, however, that the bronchial epithelium from cancer patients examined in this study may in fact be abnormally expressing erbB-2 or EGFR. Evidence suggests that some bronchial epithelium from cancer patients may have cytogenetic abnormalities (Sozzi *et al.*, 1991). In addition, other normal tissues, such as pharyngeal epithelium, have been shown to have increased expression of EGFR directly adjacent to tumour tissue (Shin *et al.*, 1994). A field effect of premalignant change in the bronchial epithelium therefore may affect erbB-2 and EGFR expression, which in turn could stimulate growth and predispose these cells to additional genetic events.

EGFR protein staining was observed only in the proliferative basal cell layer of the bronchial epithelium, while erbB-2 immunoreactivity was expressed throughout the pseudostratified architecture. Staining of EGFR in the basal layer, which is decreased in more luminal layers, suggests that this receptor may play a role in normal growth and differentiation of the bronchiolar cells. Expression of EGFR in primary tumours may vary depending on the growth state of individual cells. ErbB-2 expression may be independent of the growth or differentiated state of either bronchial epithelial cells or tumour cells since it is expressed throughout the bronchial epithelium and uniformly in tumours. A similar phenomenon was observed *in vitro* with A549 cells, in which the levels of erbB-2 mRNA were similar during active cell growth or confluence. In contrast, EGFR mRNA levels were highest in rapidly growing cells (Figure 3), and this is consistent with the higher EGFR protein levels in the proliferating basal cells of the bronchiolar epithelium.

Signal regulation by erbB-2 and EGFR is dependent on their tyrosine kinase activity. When lung adenocarcinoma tissues or cell lines were examined for activation by Western blot analysis, low-level EGFR and very little erbB-2 activation was detected. This suggests that the transforming potential of erbB-2 in lung adenocarcinomas does not involve constitutive activation of intrinsic tyrosine kinases in the absence of ligand as may occur in breast cells with amplified erbB-2 genes. Even in A549 cells, which produce both NDF and TGF-α mRNA (Figure 3a), ligands potentially capable of activating erbB-2 and EGFR respectively, only low-level erbB-2 activation is detected. Treatment of A549 and A427 cells with NDF increased the phosphotyrosine staining of the 185 kDa erbB-2 band in these cells, indicating that the receptors can be activated, however the level of activation was always less than that observed in the breast cell lines. Thus erbB-2 activation in lung adenocarcinoma tumours and cell lines is distinctly different from that of breast tissue. MCF10 benign mammary epithelial cells have low basal-level activation of erbB-2 similar to lung adenocarcinomas, but demonstrate a much greater capacity for activation by NDF. The SUM52 breast cancer cell line, which overexpresses erbB-2 protein without gene amplification, demonstrates greater basal-level activation of erbB-2 than is seen in lung adenocarcinoma cells. Amplification of erbB-2 in SKBR3 is associated with constitutive activation of greatly overexpressed erbB-2 protein. Amplification of erbB-2 expression is present in up to 30% of breast cancers and is associated with a worse patient prognosis independent of stage (Slamon *et al.*, 1987), possibly related to constitutive activation of erbB-2 tyrosine kinase. These differences between the lung and breast systems may relate to the absolute amounts of erbB-2 expressed, or may be related to the absence or presence of co-factors necessary for the activation of erbB-2 by NDF, such as erbB-3 or erbB-4 (Akita *et al.*, 1994; Plowman *et al.*, 1994) or cell specific factors (Peles *et al.*, 1993). We are currently examining whether the lung adenocarcinoma cells express either erbB-3 or erbB-4. It appears, therefore, that the role of erbB-2 in lung tumorigenesis differs from that of breast in both the frequency of amplification with constitutive activation and the capacity to be activated by NDF.

Growth assays of A549 and A427 demonstrate the capacity of these cells to grow under serum-free conditions. Autocrine growth factors NDF and TGF-α, produced by both lines, probably play a contributory role in their serum-free growth. Treatment of A549 and A427 with NDF further enhanced activation and stimulated cell growth above that of serum-free medium, demonstrating functional yet low-level activation in these cells. The relatively greater activation of EGFR relative to erbB-2 in both the lung cell lines and primary tumours, as demonstrated by Western blot staining for phosphotyrosine, may suggest that expression of erbB-2 may facilitate EGFR activation. ErbB-2 and EGFR are thought to interact by transphosphorylation via heterodimer formation, resulting in enhanced ligand affinity for the EGFR (Kokai *et al.*, 1988; Connelly *et al.*, 1990; Wada *et al.*, 1990). A549, interestingly, demonstrated an additive growth stimulus with NDF and EGF, suggesting a cooperative role between erbB-2 and EGFR, one that may exist in tumours coexpressing these receptors. Other transforming characteristics such as p53 and K-*ras* mutations are present in these cell lines (Lehman *et al.*, 1991), potentially contributing to their serum-free growth. Similarly, primary lung adenocarcinomas also contain alterations such as p53 and K-*ras* mutations (Bongiorno *et al.*, 1994), which may affect cell growth, invasive and metastatic properties. ErbB-2 and EGFR expression may therefore provide a cooperative ligand-dependent growth stimulus to bronchial epithelial cells, which acquire other critical genetic changes necessary for lung tumorigenesis.

**Acknowledgements**
The authors would like to thank Ms Cheryl Dilts for her expert assistance with the Western blot studies and Dr Jia Li for her help with lung cell cultures. This study was supported in part by a generous gift from the Norman Levy Fund.

**References**

AKITA RW, SCHAEFER GM, CARRAWAY KL, LOFGREN JA, FITZPATRICK VD, NUIJENS A, VANDLEN RL, CERIONE RA AND SLIWKOWSKI MX. (1994). A high affinity receptor for heregulin is formed from coexpression of erbB2 and erbB3. *Proc. Am. Assoc. Cancer Res.*, **35**, 43.

AMERICAN JOINT COMMITTEE ON CANCER STAGING (1992). *Manual for Staging of Cancer*, 4th edn. J.B. Lippincott: Philadelphia.

BONGIORNO PF, WHYTE RI, LESSER EJ, MOORE JH, ORRINGER MB AND BEER DG. (1994). Alterations of K-ras, p53, and erbB-2/neu in human lung adenocarcinoma. *J. Thoracic Cardiovasc. Surg.*, **107**, 590-595.

CHAZIN VR, KALEKO M, MILLER AD AND SLAMON DJ. (1992). Transformation mediated by the human HER-2 gene independent of the epidermal growth factor receptor. *Oncogene*, **7**, 1859-1866.

CONNELLY PA AND STERN D.F. (1990). The epidermal growth factor receptor and the product of the neu protooncogene are members of a receptor tyrosine phosphorylation cascade. *Proc. Natl Acad. Sci.*, **87**, 6054-6057.

CROXTALL JD, WAHEED S, CHOUDHURY Q, ANAND R AND FLOWER RJ. (1993). N-Terminal peptide fragments of lipocortin-1 inhibit A549 cell growth and block EGF-induced stimulation of proliferation. *Int. J. Cancer*, **54**, 153-158.

DAZZI H, HASLETON PS, THATCHER N, BARNES DM, WILKES S, SWINDELL R AND LAWSON RAM. (1989). Expression of epidermal growth factor receptor (EGF-R) in non-small-cell lung cancer. Use of archival tissue and correlation of EGF-R with histology, tumour size, node status and survival. *Br. J. Cancer*, **59**, 746-749.

DERYNCK R, ROBERTS AB, WINKLER ME, CHEN EY, GOEDDEL DV. (1984). Human transforming growth factor-alpha: precursor structure and expression in E. coli. *Cell*, **38**, 287-297.

DI FIORE PP, PIERCE JH, FLEMMING TP, HAZEN R, ULLRICH A, KING CR, SCHLESSINGER J AND AARONSON SA. (1987). erbB2 is a potent oncogene when over-expressed in NIH3T3 cells. *Science*, **237**, 178-182.

DIATTADI R, GION M, PAGAN V., BRAZZALE A, DEL MASCHIO O, BARGOSSI A, BUSETTO A AND BRUSCAGNIN G. (1991). Epidermal growth factor receptor in lung malignancies. Comparison between cancer and normal tissue. *Br. J. Cancer*, **64**, 741-744.

DI MARCO E, PIERCE JH, FLEMING TP, KRAUS MH, MOLLOY CJ, AARONSON SA AND DI FIORE PP. (1989). Autocrine interaction between TGFα and the EGF-receptor: quantitative requirements for induction of the malignant phenotype. *Oncogene*, **4**, 831-838.

ETHIER SP, MAHACEK ML, GULLICK WJ, FRANK TS AND WEBER BL. (1993). Differential isolation of normal luminal mammary epithelial cells and breast cancer cells from primary and metastatic sites using selective media. *Cancer Res.*, **53**, 627-635.

HANSON LA, NUZUM EO, JONES BC, MALKINSON AM AND BEER DG. (1991). Expression of the glucocorticoid receptor and K-ras genes in urethane induced mouse lung tumours and transformed cell lines. *Exp. Lung Res.*, **17**, 371-387.

HOLMES WE, SLIWKOWSKI MX, AKITA RW, HENZEL WJ, LEE J, PARK JW, YANSURE D, ABADI N, RAAB H, LEWIS GD, SHEPARD HM, KUANG WJ, WOOD WI, GOEDDEL DV AND VANDLEN RL. (1992). Identification of Heregulin, a specific activator of $p185^{erbB2}$. *Science*, **256**, 1205-1210.

KERN JA, SCHWARTZ DA, NORDBERG JE, WEINER DB, GREENE MI, TORNEY L AND ROBINSON RA. (1990). $P185^{neu}$ Expression in human lung adenocarcinomas predicts shortened survival. *Cancer Res.*, **50**, 5184-5191.

KOKAI Y, DOBASHI K, WEINER DB, MYERS JN, NOWELL PC AND GREENE MI. (1988). Phosphorylation process induced by epidermal growth factor alters the oncogenic and cellular *neu* (NGL) gene products. *Proc. Natl Acad. Sci. USA.*, **85**, 5389-5393.

LEHMAN TA, BENNETT WP, METCALF RA, WELSH JA, ECKER J, MODALI RV, ULLRICH S, ROMANO JW, APPELLA E, TESTA JR, GERWIN BI AND HARRIS CC. (1991). p53 mutations, rat mutations, and p53-heat shock 70 protein complexes in human lung carcinoma cell lines. *Cancer Res.*, **51**, 4090-4096.

MADTES DK. (1993). Transforming growth factor-α and epidermal growth factor. In *Cytokines of the Lung*, Kelley J. (ed.) pp. 139-181, Marcel-Dekker: New York.
NOGUCHI M, MURAKAMI M, BENNETT W, LUPU R, HUI F, HARRIS CC AND GERWIN BI. (1993). Biological consequences of overexpression of a transfected c-erbB-2 gene in immortalized human bronchial epithelial cells. *Cancer Res.*, **53**, 2035-2043.
OSAKI A, TOI M, YAMADA H, KAWAMI H, KUROI K AND TOGE T. (1992). Prognostic significance of co-expression of c-erbB-2 oncoprotein and epidermal growth factor receptor in breast cancer patients. *Am. J. Surg.*, **164**, 323-326.
PAVELIC K, BANJAC Z, PAVELIC J AND SPAVENTI S. (1993). Evidence for a role of EGF receptor in the progression of human lung carcinoma. *Anticancer Res.*, **13**, 1133-1138.
PELES E, BEN-LEVY R, TZAHAR E, LIU N, WEN D AND YARDEN Y. (1993). Cell-type specific interaction of Neu differentiation factor (NDF/heregulin) with Neu/HER-2 suggests complex ligand-receptor relationships. *EMBO J.*, **12**, 961-971.
PLOWMAN GD, GREEN JM, CULOUSCOU JM, CARLTON GW, ROTHWELL VM AND BUCKLEY S. (1993). Heregulin induces tyrosine phosphorylation of HER4/p180$^{erbB4}$. *Nature*, **366**, 473-475.
PRESS MF, CORDON-CARDO C AND SLAMON DJ. (1990). Expression of the HER-2/neu proto-oncogene in normal human adult and fetal tissues. *Oncogene*, **5**, 953-962.
PRESS MF, HUNG G, GODOLPHIN W AND SLAMON DF. (1994). Sensitivity of HER-2/neu antibodies in archival tissue samples: Potential source of error in immunohistochemical studies of oncogene expression. *Cancer Res.*, **54**, 2771-2777.
RUSCH V, BASELGA J, CORDON-CARDO C, ORAZEM J, ZAMAN M, HODA S, McINTOSH J, KURIE J AND DMITROVSKY E. (1993). Differential expression of the epidermal growth factor receptor and its ligands in primary non-small cell lung cancers and adjacent benign lung. *Cancer Res*, **53**, 2379-2385.
SCAGLIOTTI,. GV, LEONARDO E, CAPPIA S, MASIERO P, MICELA M, GUBETTA L AND POZZI E. (1993). Epidermal growth factor receptor and neu-oncogene expression in lung cancer. *Proc. Am. Soc. Clin. Oncol.*, **12**, A1090.
SEGATTO O, LONARDO F, PIERCE JH, BOTTARO DP AND DI FOIRE PP. (1990). The role of autophosphorylation in modulation of erbB-2 transforming function. *New Biol.*, **2**, 187-195.
SHIN DM, RO JY, HONG WK AND HITTELMAN WN. (1994). Dysregulation of epidermal growth factor receptor expression in premalignant lesions during head and neck tumorigenesis. *Cancer Res*, **54**, 3153-3159.
SLAMON DJ, CLARK GM, WONG SG, LEVIN WJ, ULLRICH A AND McGUIRE WL. (1987). Human breast cancer: Correlation of relapse and survival with amplification of the HER-2/neu Oncogene. *Science*, **235**, 177-182.
SLAMON DJ, GODOLPHIN W, JONES LA, HOLT JA, WONG SG, KEITH DE, LEVIN WJ, STUART SG, UDOVE J, ULLRICH A AND PRESS MF. (1989). Studies of the HER-2/neu proto-oncogene in human breast and ovarian cancer. *Science*, **244**, 707-712.
SOZZI G, MIOZZO M, TAGLIABUE E, CALDERONE C, LOMBARDI L, PILOTTI S, PASTORINO U, PIEROTTI MA AND PORTA GD. (1991). Cytogenetic abnormalities and overexpression of receptors for growth factors in normal bronchial epithelium and tumor samples of lung cancer patients. *Cancer Res.*, **51**, 400-404.
SPEIRS V, RAY KP AND FRESHNEY RI. (1991). Paracrine control of differentiation in the alveolar carcinoma, A549, by human foetal lung fibroblasts. *Br. J. Cancer*, **64**, 693-699.
STOCKLIN E, BOTTERI F AND GRONER B. (1993). An activated allele of the c-erbB-2 oncogene impairs kidney and lung function and causes early death of transgenic mice. *J. Cell Biol.*, **122**, 199-208.
SUDA Y, AIZAWA S, FURUTA Y, YAGI T, IKAWA Y, SAITOH K, YAMADA Y, TOYOSHIMA K AND YAMAMOTO T. (1990). Induction of a variety of tumors by c-erbB2 and clonal nature of lymphomas even with mutated gene ($Val^{659}$-$Glu^{659}$). *EMBO J.*, **9**, 181-190.
TATEISHI M, ISHIDA T, MITSUDOMI T, KANEKO S AND SUGIMACHI K. (1990). Immunohistochemical evidence of autocrine growth factors in adenocarcinoma of the human lung. *Cancer Res.*, **50**, 7077-7080.
VEALE D, KERR N, GIBSON GJ, KELLY PJ AND HARRIS AL. (1993). The relationship of quantitative epidermal growth factor receptor expression in non-small cell lung cancer to long term survival. *Br. J. Cancer*, **68**, 162-165.
VELU TJ, BEGUINOT L, VASS WC, WILLINGHAM MC, MERLINO GT, PASTAN I AND LOWRY DR. (1987). Epidermal growth factor-dependent transformation by a human EGF receptor protooncogene. *Science*, **238**, 1408-1410.
WADA T, QUAN X AND GREENE MI. (1990). Intermolecular association of the p185$^{neu}$ protein and EGF receptor modulates EGF receptor function. *Cell*, **61**, 1339-1347.
WEINER DB, NORDBERG J, ROBINSON R, NOWELL PC, GAZDAR A, GREENE MI, WILLIAMS WV, COHEN JA AND KERN JA. (1990). Expression of the neu gene-encoded protein (p185$^{neu}$) in human non-small cell carcinoma of the lung. *Cancer Res.*, **50**, 421-425.
XU YH, ISHII S, CLARK AJ, SULLIVAN M, WILSON RK, MA DP, ROE BA, MERLINO GT AND PASTAN I. (1984). Human epidermal-growth-factor-receptor cDNA is homologous to a variety of RNAs over-produced in A431 carcinoma cells. *Nature*, **309**, 806-810.
YU D, WANG SS, DULSKI KM, TSAI CM, NICOLSON GL AND HUNG MC. (1994). c-erbB-2/neu overexpression enhances metastatic potential of human lung cancer cells by induction of metastasis-associated properties. *Cancer Res.*, **54**, 3260-3266.